# Fludrocortisone Induces Aortic Pathologies in Mice

**DOI:** 10.3390/biom12060825

**Published:** 2022-06-13

**Authors:** Dien Ye, Congqing Wu, Hui Chen, Ching-Ling Liang, Deborah A. Howatt, Michael K. Franklin, Jessica J. Moorleghen, Samuel C. Tyagi, Estrellita Uijl, A. H. Jan Danser, Hisashi Sawada, Alan Daugherty, Hong S. Lu

**Affiliations:** 1 Saha Cardiovascular Research Center, Lexington, KY 40536, USA; d.ye@erasmusmc.nl (D.Y.); cwu3@uky.edu (C.W.); hui.chen@uky.edu (H.C.); jenny210217@uky.edu (C.-L.L.); ddhowa2@uky.edu (D.A.H.); michael.franklin@uky.edu (M.K.F.); jjmoorl@uky.edu (J.J.M.); sam.tyagi@uky.edu (S.C.T.); hisashi.sawada@uky.edu (H.S.); alan.daugherty@uky.edu (A.D.); 2 Division of Pharmacology and Vascular Medicine, Department of Internal Medicine, Erasmus University Medical Center, 3015 CN Rotterdam, The Netherlands; e.uijl@erasmusmc.nl (E.U.); a.danser@erasmusmc.nl (A.H.J.D.); 3 Saha Cardiovascular Research Center, Department of Surgery, College of Medicine, University of Kentucky, Lexington, KY 40536, USA; 4 Department of Physiology, University of Kentucky, Lexington, KY 40536, USA; 5 Saha Aortic Center, University of Kentucky, Lexington, KY 40536, USA

**Keywords:** aortic aneurysms, aortic dissection, fludrocortisone, angiotensin, hypercholesterolemia, mouse

## Abstract

Background and Objective: In an experiment designed to explore the mechanisms of fludrocortisone-induced high blood pressure, we serendipitously observed aortic aneurysms in mice infused with fludrocortisone. The purpose of this study was to investigate whether fludrocortisone induces aortic pathologies in both normocholesterolemic and hypercholesterolemic mice. Methods and Results: Male adult C57BL/6J mice were infused with either vehicle (85% polyethylene glycol 400 (PEG-400) and 15% dimethyl sulfoxide (DMSO); *n* = 5) or fludrocortisone (12 mg/kg/day dissolved in 85% PEG-400 and 15% DMSO; *n* = 15) for 28 days. Fludrocortisone-infused mice had higher systolic blood pressure, compared to mice infused with vehicle. Fludrocortisone induced aortic pathologies in 4 of 15 mice with 3 having pathologies in the ascending and aortic arch regions and 1 having pathology in both the ascending and descending thoracic aorta. No pathologies were noted in abdominal aortas. Subsequently, we infused either vehicle (*n* = 5/group) or fludrocortisone (*n* = 15/group) into male ApoE ^−/−^ mice fed a normal laboratory diet or LDL receptor ^−/−^ mice fed either normal or Western diet. Fludrocortisone increased systolic blood pressure, irrespective of mouse strain or diet. In ApoE ^−/−^ mice infused with fludrocortisone, 2 of 15 mice had ascending aortic pathologies, but no mice had abdominal aortic pathologies. In LDL receptor ^−/−^ mice fed normal diet, 5 had ascending/arch pathologies and 1 had pathologies in the ascending, arch, and suprarenal aortic regions. In LDL receptor ^−/−^ mice fed Western diet, 2 died of aortic rupture in either the descending thoracic or abdominal region, and 2 of the 13 survived mice had ascending/arch aortic pathologies. Aortic pathologies included hemorrhage, wall thickening or thinning, or dilation. Only ascending aortic diameter in LDLR ^−/−^ mice fed Western diet reached statistical significance, compared to their vehicle. Conclusion: Fludrocortisone induces aortic pathologies independent of hypercholesterolemia. As indicated by the findings in mouse studies, people who are taking or have taken fludrocortisone might have an increased risk of aortic pathologies.

## 1. Introduction

Fludrocortisone is a corticosteroid used to improve sodium and water balance under certain conditions such as adrenocortical insufficiency [1]. It is also frequently used in patients with orthostatic hypotension because fludrocortisone can raise blood pressure [2]. However, the mechanism of increased blood pressure is not fully understood.

The renin–angiotensin system regulates blood pressure and water and sodium homeostasis [3]. Angiotensinogen (AGT) is the unique substrate for angiotensin II (AngII), the major bioactive peptide in this hormonal system. Its deletion, e.g., by small interfering RNA (siRNA) targeted to liver AGT, results in lower blood pressure [4]. siRNA-mediated blood pressure reduction in case of emergency was reversed by fludrocortisone in rats [4]. We evaluated fludrocortisone in hepatocyte-specific AGT deficient (hepAGT ^−/−^) mice or their wild type (hepAGT +/+) littermates (Ye et al., unpublished data). One mouse infused with fludrocortisone died before termination. Necropsy showed that the mouse had aortic rupture in the suprarenal aortic region, a common location for AngII-induced aortic rupture in mice [5,6,7,8]. During termination, we dissected and characterized aortas from both hepAGT +/+ and ^−/−^ mice. We found that some mice infused with fludrocortisone exhibited aortic pathologies in either or both ascending/arch and abdominal aortic regions, comparable to what we see in AngII-infused mice [9].

HepAGT ^−/−^ mice and their wild type littermates were developed initially in a mixed 129/C57BL/6N background [10,11], then backcrossed to C57BL/6J strain six times. Our observations were made in a low-density lipoprotein receptor (LDLR ^−/−^) mouse strain. It is unclear whether the effect of fludrocortisone on aortic pathology is related to hypercholesterolemia or specific strains. AngII-induced abdominal aortic aneurysms (AAA) are augmented under hypercholesterolemic conditions such as LDLR deficiency or apolipoprotein E (ApoE) deficiency, whereas AngII-induced thoracic aortic aneurysms (TAA) are not associated with hypercholesterolemia [12,13]. Therefore, in this study, we determined whether fludrocortisone induces aortic aneurysms in mice with C57BL/6J background and whether the incidence of aortic pathologies is different between normocholesterolemic and hypercholesterolemic mice.

## 2. Materials and Methods

### 2.1. Mice

Male C57BL/6J mice, LDLR ^−/−^ mice, and ApoE ^−/−^ mice were purchased from The Jackson Laboratory (Table 1). Only male mice were studied because female mice have a low incidence of both AAA and TAA compared to male mice, as reported in several mouse models [14]. Mice were fed Teklad Irradiated Global 18% Protein Rodent Diet #2918 (Envigo, Indianapolis, IN, USA) and given access to water ad libitum. In one study, LDLR ^−/−^ mice were fed a Western diet (Diet # TD.88137, ENVIGO) for 1 week before the vehicle or fludrocortisone was infused, and this special diet was continued for another 4 weeks during vehicle or fludrocortisone infusion.

**Figure 1:**
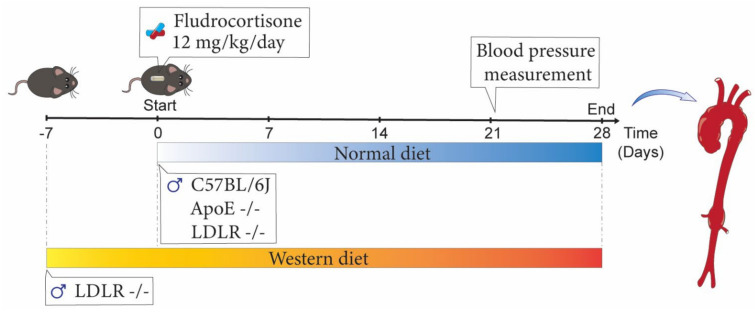
Timeline of fludrocortisone infusion studies. Adult male C57BL/6J, ApoE ^−/−^, or LDLR ^−/−^ mice were infused with either vehicle or fludrocortisone for 28 days. C57BL/J mice and ApoE ^−/−^ mice were fed normal diet. LDLR ^−/−^ mice were either fed normal or Western diet.

All animal experiments (timeline shown in Figure 1) reported in this manuscript were performed with the approval of the University of Kentucky Institutional Animal Care and Use Committee (IACUC protocol number 2018-2968) and followed the ARRIVE Guidelines (Table 2) [15].

### 2.2. Mini Osmotic Pump Implantation and Fludrocortisone Infusion

Fludrocortisone (Cat# F6127-1G; MilliporeSigma, St. Louis, MO, USA) was infused subcutaneously via mini osmotic pumps (Alzet Model 2004; Durect Corp., Cupertino, CA, USA). Polyethylene glycol-400 (PEG-400; 85% vol/vol; Cat# PX1286-B2; MilliporeSigma) and DMSO (15% vol/vol; Cat# D8418-50ML; MilliporeSigma) were used to dissolve fludrocortisone. Therefore, PEG-400 (85% *vol*/*vol*) and DMSO (15% *vol*/*vol*) without fludrocortisone were used as vehicle.

Male mice at 8–9 weeks of age were infused with either vehicle or fludrocortisone (12 mg/kg/day) for 4 weeks. Mice were sedated with isoflurane, and pumps were implanted subcutaneously on the right flank of each mouse in the same procedure described for AngII infusion [9].

### 2.3. Systolic Blood Pressure Measurements

Systolic blood pressure was measured daily (between 1–5 PM) on days 21–23 using a non-invasive tail-cuff system (BP-2000 blood pressure analysis system; Visitech Systems, Inc., Apex, NC, USA) following our standard protocol [16]. Considering that many factors may affect blood pressure measurements using this tail-cuff system, we did not compare data that were measured on different dates between different mouse strains.

### 2.4. Plasma Collection and Total Cholesterol Measurement

Mice were anesthetized using ketamine/xylazine cocktail at termination. Blood samples were harvested by right ventricular puncture with EDTA (final concentration: 1.8 mg/mL) and then centrifuged at 400 g × 20 min, 4 °C to prepare plasma. Plasma cholesterol concentrations were measured using an enzymatic kit (Cat # C7510; Pointe Scientific Inc., Canton, MI, USA).

### 2.5. Measurement of Aortic Diameters

Immediately after euthanasia, saline was perfused through the left ventricle to remove the blood in the aorta. The organs and tissues surrounding the ascending, aortic arch, and proximal descending thoracic regions were removed carefully. A thin black plastic sheet was placed behind the heart and thoracic aorta, and in situ images were captured using a Nikon dissecting stereoscope [17,18]. Aortic diameters were measured at the largest width perpendicular to the aortic longitudinal axis at the ascending aorta using a Nikon NIS-Elements AR 4.51.

Subsequently, the full length of the aorta from the ascending portion to the iliac bifurcation was dissected and fixed in 10% neutral-buffered formalin for 24 h. Adventitia were then removed, and aortas were pinned and imaged. Maximal outer width of the suprarenal aortic region was measured using the Nikon NIS-Elements AR 4.51.

### 2.6. Statistical Analyses

Individual data are presented with the median and 25th/75th percentiles, and the whiskers represent 5% and 95% intervals. Statistical analyses were performed using SigmaPlot version 14.5 (SYSTAT Software Inc., San Jose, CA, USA). Since *n* = 5 for each vehicle group (small sample size), only Mann–Whitney rank sum test was used for data analyses of plasma total cholesterol concentrations, systolic blood pressure, and aortic diameters. The correlation between ascending aortic diameters and systolic blood pressure was analyzed using Spearman rank order correlation analysis. *p* < 0.05 was considered statistically significant.

## 3. Results

### 3.1. Fludrocortisone Induced Aortic Pathologies in the Thoracic Aorta of Male C57BL/6J Mice

**Figure 2:**
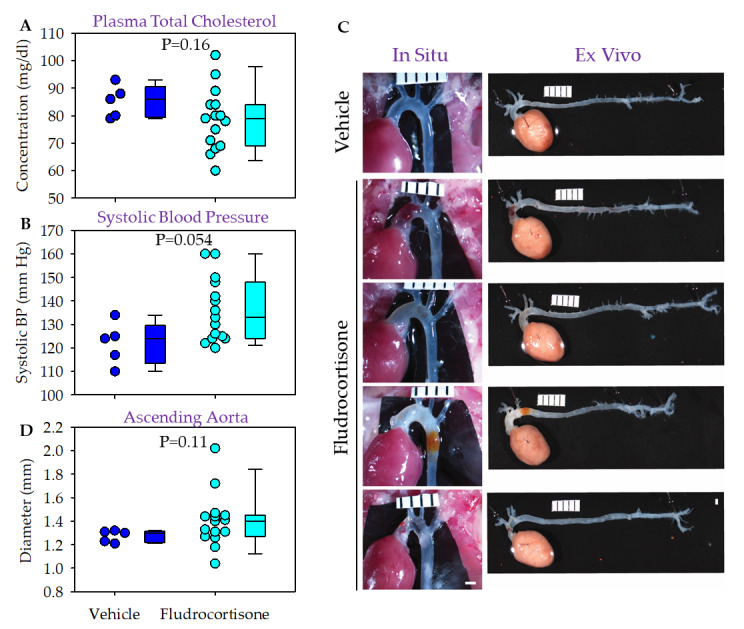
Fludrocortisone induced aortic pathologies in male C57BL/6J mice. (**A**) Plasma total cholesterol concentrations were measured using an enzymatic kit. (**B**) Systolic blood pressure was measured using a tail-cuff system. (**C**) In situ and ex vivo images were taken using a Nikon SMZ800 stereoscope. (**D**) Maximal outer diameter of the ascending aorta was measured using NIS-Elements AR 4.51 software (Nikon). Scale bar = 1 mm.

Male C57BL/6J mice were fed a normal laboratory diet and infused with either vehicle or fludrocortisone for 4 weeks. Plasma cholesterol concentrations were below 100 mg/dL for both groups (Figure 2A). Fludrocortisone infusion led to higher systolic blood pressure in male C57BL/6J mice (Figure 2B) compared to mice infused with vehicle, but this did not reach statistical significance (*p* = 0.054). No mice died or were excluded during the study. No pathologies were noted in mice infused with vehicle per in situ and ex vivo imaging analysis. In mice infused with fludrocortisone, 4 of 15 mice had gross pathological changes in the thoracic aortic regions, but no pathology was detected in abdominal regions (Figure 2C). One mouse showed extensive hemorrhage and another mouse showed modest hemorrhage in the ascending aortic region. One mouse had profound dilation in the ascending aortic region, and one mouse had dilation, wall thickening, and restricted thinning in the ascending region and discoloration in the descending aorta, possibly due to the hemorrhage resolution.

We measured the maximal diameter of ascending (Figure 2D), descending thoracic (data not shown), and suprarenal abdominal aortic regions (median: 0.8 versus 0.9 mm, *p* = 0.001). No significant differences in diameters of the ascending and descending thoracic aortas, respectively, were detected.

### 3.2. Fludrocortisone Induced Aortic Pathologies in Male ApoE ^−/−^ Mice and LDLR ^−/−^ Mice

**Figure 3:**
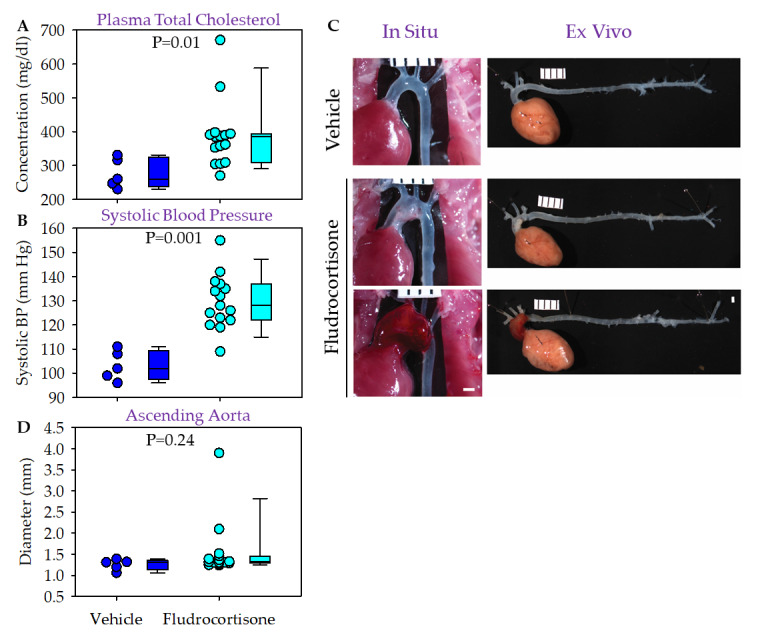
Fludrocortisone induced aortic pathologies in male ApoE ^−/−^ mice. (**A**) Plasma total cholesterol concentrations were measured using an enzymatic kit. (**B**) Systolic blood pressure was measured using a tail-cuff system. (**C**) In situ and ex vivo images were taken using a Nikon SMZ800 stereoscope. (**D**) Maximal outer diameter of the ascending aorta was measured using NIS-Elements AR 4.51 software (Nikon). Scale bar = 1 mm.

AAA, but not TAA, is augmented by hypercholesterolemia in AngII-infused male mice [12,13]. Therefore, we determined whether hypercholesterolemia augments fludrocortisone-induced aortic pathologies in the two commonly used hypercholesterolemic mouse models, ApoE ^−/−^ mice and LDLR ^−/−^ mice. ApoE ^−/−^ mice become hypercholesterolemic spontaneously even when fed a normal laboratory rodent diet [19]. In male ApoE ^−/−^ mice fed a normal laboratory diet, although both groups were modestly hypercholesterolemic, the median plasma cholesterol concentration in the fludrocortisone group was higher than that in the vehicle group (Figure 3A). No death was found and no severe health condition led to withdrawal of any study mouse. Therefore, all study mice were included for blood pressure, imaging, and diameter analysis.

Fludrocortisone increased systolic blood pressure (Figure 3B) compared to the vehicle infusion. Among the 15 mice infused with fludrocortisone, one had profound dilation in the ascending and arch regions, and one had a large fresh hemorrhage in the ascending and arch regions (Figure 3C). No mice had apparent abdominal aortic pathologies (Figure 3C). We did not detect significant differences in aortic diameters in the ascending (Figure 3D), descending thoracic (data not shown), and suprarenal aortic regions (data not shown) between vehicle and fludrocortisone-infused mice.

In contrast to the spontaneous hypercholesterolemia in ApoE ^−/−^ mice, LDLR ^−/−^ mice have only modestly higher plasma total cholesterol concentrations than C57BL/6J mice when fed normal laboratory diet, but their plasma cholesterol concentrations increase profoundly when fed a saturated fat-enriched diet. Therefore, we performed two separate studies to determine the effects of fludrocortisone on aortic pathologies in male LDLR ^−/−^ mice.

**Figure 4:**
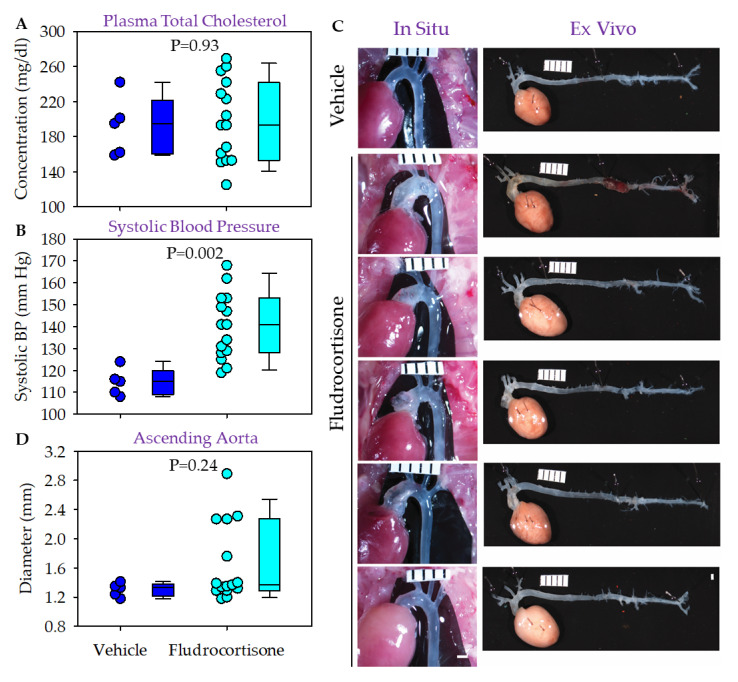
Fludrocortisone induced aortic pathologies in male LDLR ^−/−^ mice fed normal diet. (**A**) Plasma total cholesterol concentrations were measured using an enzymatic kit. (**B**) Systolic blood pressure was measured using a tail-cuff system. (**C**) In situ and ex vivo images were taken using a Nikon SMZ800 stereoscope. (**D**) Maximal outer diameter of the ascending aorta was measured using NIS-Elements AR 4.51 software (Nikon). Scale bar = 1 mm.

In the first study, we fed male LDLR ^−/−^ mice normal laboratory rodent diet. Medians of plasma cholesterol concentrations were not different between the two groups (Figure 4A). The systolic blood pressure increase was significant in mice infused with fludrocortisone, compared to mice infused with vehicle (Figure 4B). There was no death prior to termination or exclusion due to other reasons. Among the 15 mice infused with fludrocortisone, five had apparent ascending aortic dilation with a grossly thin or thick wall (Figure 4C). Among the five mice, one mouse also had aortic pathology with a relatively fresh hemorrhage in the suprarenal region (Figure 4C). We did not find significant differences in aortic diameters in the ascending (Figure 4D), descending thoracic (data not shown), and suprarenal aortic regions (data not shown) between the two groups. 

**Figure 5:**
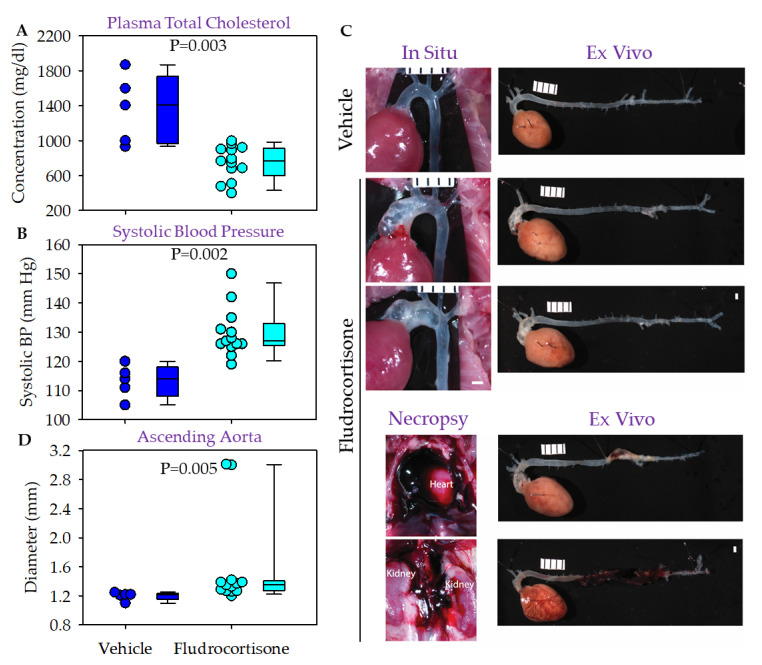
Fludrocortisone induced aortic pathologies in male LDLR ^−/−^ mice fed Western diet. (**A**) Plasma total cholesterol concentrations were measured using an enzymatic kit. (**B**) Systolic blood pressure was measured using a tail-cuff system. (**C**) In situ and ex vivo images were taken using a Nikon SMZ800 stereoscope. (**D**) Maximal outer diameter of the ascending aorta was measured using NIS-Elements AR 4.51 software (Nikon). Scale bar = 1 mm.

In the subsequent study, we fed male LDLR ^−/−^ mice a Western diet containing 42% kcal/kcal from fat for 1 week. Fludrocortisone or vehicle was then infused for 28 days, while the Western diet feeding was continued. Two of 15 mice died of aortic rupture. These two mice were excluded from plasma cholesterol, blood pressure, and aortic diameter data analyses. Although both groups were hypercholesterolemic, plasma cholesterol concentrations were lower in mice infused with fludrocortisone than in those infused with vehicle (Figure 5A). Systolic blood pressure was higher in mice infused with fludrocortisone, compared to those infused with vehicle (Figure 5B). Among the surviving 13 mice, two had striking dilations and thinned wall in the ascending and aortic arch regions (Figure 5C). For the two mice with aortic rupture, one rupture initiated in the ascending thoracic aortic region, and one occurred in the suprarenal aortic region. No mice had abdominal aortic pathology except the one that died of abdominal aortic rupture. The median of the ascending aortic diameter was larger in fludrocortisone-infused mice than in vehicle-infused mice (Figure 5D). No significant differences in diameters of descending thoracic and abdominal regions were detected between the two groups (data not shown).

**Figure 6:**
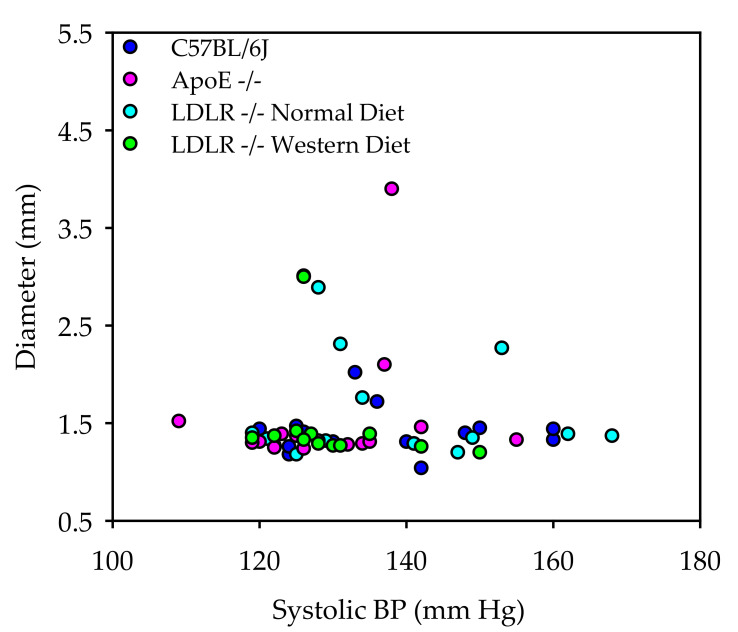
Ascending aortic diameters were not associated with systolic blood pressure in mice infused with fludrocortisone. Colors represent different mouse strains. Spearman Rank Order Correlation analysis shows *p* > 0.05.

Aortic pathologies are summarized in Table 3. We also performed correlation analysis to determine whether higher systolic blood pressure is associated with fludrocortisone-induced aortic dilation (Figure 6). No significant associations between systolic blood pressure and ascending aortic diameters were detected in the three mouse strains fed normal diet or in LDLR ^−/−^ mice fed Western diet.

## 4. Discussion

The present study determined effects of fludrocortisone on aortic pathologies in four independent experiments using three mouse strains (C57BL6/J, ApoE ^−/−^, and LDLR ^−/−^ mice fed either normal laboratory or Western diet). Although the incidence of aortic pathology was relatively low (15 of 60 = 25%), it was evident that fludrocortisone contributed to aortic pathologies, irrespective of mouse strain or diet.

Aortic pathologies were found predominantly in the ascending and aortic arch regions. Among the 15 mice with profound aortic pathologies, 14 were involved in the ascending and aortic arch regions, which included two having both ascending and descending thoracic aortic pathologies. One mouse died of aortic rupture with descending thoracic and abdominal aortic hemorrhage, and one died of abdominal aortic rupture. Aortic pathologies in the ascending and aortic arch regions include dilatation, thin or thick wall, and hemorrhage. These pathologies were similar to those observed in an AngII-infused mouse model [13].

Fludrocortisone induced abdominal aortic pathology locates in the suprarenal aorta, the same location for AngII-induced AAAs [5,6,9] mice with endothelial nitric oxide synthase [20], mice administered deoxycorticosterone acetate and high salt, and mice administered aldosterone and high salt [21]. The mechanism of this specific location for AAAs in mouse models is unclear.

In C57BL/6J or LDLR ^−/−^ mice fed normal laboratory diet, plasma cholesterol concentrations were not different between the two groups. In ApoE ^−/−^ mice fed normal diet, plasma cholesterol concentrations were modestly higher in mice infused with fludrocortisone. In contrast, in LDLR ^−/−^ mice fed Western diet, plasma cholesterol concentrations were lower in mice infused with fludrocortisone than in mice infused with vehicle. There is compelling evidence from our own research work and that of others [12,19,22] that ApoE ^−/−^ mice have barely detectable HDL in plasma, while C57BL/6J mice have predominant HDL in plasma. Western diet feeding leads to profound increases in non-HDL lipoproteins in LDLR ^−/−^ mice, whereas normal rodent diet feeding shows low non-LDL lipoproteins in this mouse strain. Despite the different distributions of plasma lipoproteins, we did not observe different incidence or severity of aortic pathologies in the three mouse strains or in LDLR ^−/−^ mice fed normal versus Western diet. Additionally, we did not find that plasma cholesterol concentrations correlated with aortic pathologies in fludrocortisone-infused mice. In contrast to our findings in mice infused with fludrocortisone, it is well-established that AngII-induced AAAs are augmented by hypercholesterolemia [12]. Therefore, it is likely that the molecular mechanisms underlying fludrocortisone-induced versus AngII-induced abdominal aortic pathologies are different. Nevertheless, we acknowledge that one limitation of the present study is that we do not know whether fludrocortisone changed lipoprotein distributions. Moreover, we were unable to find previous reports that fludrocortisone affected plasma total cholesterol concentrations or lipoprotein distributions in either animal models or humans.

Similarly to AngII, fludrocortisone increased systolic blood pressure in mice. It appears that the magnitude of the increase in fludrocortisone-infused mice is less than in AngII-infused mice. However, conclusions cannot be made because we did not perform side-by-side comparisons between these two reagents. There is compelling evidence that AngII-induced aortic pathologies are independent of blood pressure changes [23,24,25]. Of note, norepinephrine can also increase blood pressure by the same magnitude as that of AngII, but no aortic aneurysms were detected in mice infused with norepinephrine [24,25]. We compared the blood pressure in mice exhibiting aortic pathologies with those without aortic pathologies. We did not note apparent associations between blood pressure and aortic pathologies. Based on these observations, it seems reasonable to conclude that high blood pressure is not a primary contributing factor to aortic pathologies in fludrocortisone-infused mice.

Fludrocortisone is a drug commonly used in patients with adrenocortical insufficiency [1], orthostatic hypotension [2], and some other conditions. This drug has many known adverse effects, but most are mild. No studies have reported that fludrocortisone induces aortic pathologies in humans. In fact, there are studies that have reported that fludrocortisone treatment can improve efficiency of hypervolemic therapy in patients with aneurysmal subarachnoid hemorrhage [26,27,28]. It is unclear whether our findings are species-specific.

Fludrocortisone is a mineralocorticoid receptor agonist. One study reported that deoxycorticosterone acetate (DOCA) or aldosterone, in the presence of high salt in drinking water, induced aortic aneurysms in C57BL/6 mice that mimicked the phenotypes in mice infused with AngII [21]. Aortic aneurysms induced by either DOCA or aldosterone and high salt were attenuated by mineralocorticoid receptor antagonists [21]. A recent prospective human study reported that patients with primary aldosteronism had larger ascending aortas [29]. Together, these findings support the notion that mineralocorticoid receptor may play a role in the development of aortic aneurysms.

Considering that human and mouse aortic pathologies have many comparable pathogeneses, it is worth paying attention to possible aortic pathologies in patients who are taking fludrocortisone. It is also important to be cautious when prescribing fludrocortisone to patients with aortic pathologies—our findings suggest that these patients should be at least monitored more rigorously during fludrocortisone administration.

In conclusion, this study provides evidence that fludrocortisone induces aortic pathologies in mice, predominantly in the ascending and arch regions, with risk for rupture in both the thoracic and abdominal aortic regions. Fludrocortisone-induced aortic pathologies were not attributed to mouse strain, hypercholesterolemia, or blood pressure.

## Figures and Tables

**Table 1:** Mouse Strain Information.

Strain	Description	Sex	Persistent ID	URL
C57BL/6J	C57BL/6J	M	000664	https://www.jax.org/strain/000664 (accessed on 22 May 2022)
ApoE ^−/−^	B6.129P2-*Apoe^tm1Unc^*/J (>N10 to C57BL/6J)	M	002052	https://www.jax.org/strain/002052 (accessed on 22 May 2022)
LDLR ^−/−^	B6.129S7-*Ldlr^tm1Her^*/J (>N10 to C57BL/6J)	M	002207	https://www.jax.org/strain/002207 (accessed on 22 May 2022)

**Table 2:** ARRIVE Guidelines Checklist.

Item	Application
IACUC Protocol	# 2018-2968, approved by the University of Kentucky IACUC.
Sex	This study examined aortic pathologies only in male mice because of the higher prevalence in male mice [14].
Inclusion criteria	Body weight > 20 g and 7–9 weeks of age
Exclusion criteria	Body weight < 20 g and <7 or >9 weeks of age Euthanasia or death prior to termination or medical cases reported by a veterinarian were excluded for in situ and ex vivo diameter measurements
Sample size	*n* = 5 for vehicle, and *n* = 15 for fludrocortisone infusion in each experiment
Power analysis (prospective)	Not performed
Endpoint	Death due to aortic rupture Aortic diameters by in situ or ex vivo measurements in survived animals
Randomization	DLAR staff placed study mice randomly in cages (*n* = 5/cage) upon arrival.
Blinding	All experimental data were verified by an independent investigator blinded to the study group information.
Statistical analysis	SigmaPlot version 14.5 (SYSTAT Software Inc., Palo Alto, CA, USA)
Statistical method	Continuous variables between groups were analyzed using Mann–Whitney rank sum test. The correlation between ascending aortic diameters and systolic blood pressure was analyzed using Spearman rank order correlation analysis.
Data availability	All raw data and analytical methods are available from the corresponding authors upon appropriate request.

**Table 3:** Summary of Aortic Pathologies.

Mouse Strain	Diet	Aortic Pathologies (*n* of Mice)			
		Asc/Arch	Asc/Arch + Desc	Asc/Arch + Suprarenal	Desc + Suprarenal
C57BL/6J	Normal	3	1	0	0
ApoE ^−/−^	Normal	2	0	0	0
LDLR ^−/−^	Normal	4	0	1	0
LDLR ^−/−^	Western	2	0	1	1

Asc = ascending aorta; Arch = aortic arch; Desc = descending thoracic.

## Data Availability

Detailed Materials and Methods are presented in the Methods section. The raw data that support the findings reported in this manuscript are available from the corresponding author upon reasonable request.
